# Characterizing the impact of sustained sulfadoxine/pyrimethamine use upon the *Plasmodium falciparum* population in Malawi

**DOI:** 10.1186/s12936-016-1634-6

**Published:** 2016-11-29

**Authors:** Matt Ravenhall, Ernest Diez Benavente, Mwapatsa Mipando, Anja T. R. Jensen, Colin J. Sutherland, Cally Roper, Nuno Sepúlveda, Dominic P. Kwiatkowski, Jacqui Montgomery, Kamija S. Phiri, Anja Terlouw, Alister Craig, Susana Campino, Harold Ocholla, Taane G. Clark

**Affiliations:** 1 Faculty of Infectious and Tropical Diseases, London School of Hygiene and Tropical Medicine, London, UK; 2 Department of Physiology, College of Medicine, University of Malawi, Blantyre, Malawi; 3 Centre for Medical Parasitology, University of Copenhagen, Copenhagen, Denmark; 4 Centre for Statistics and Applications of University of Lisbon, Lisbon, Portugal; 5 Wellcome Trust Sanger Institute, Hinxton, UK; 6 Liverpool School of Tropical Medicine, Pembroke Place, Liverpool, UK; 7 Malawi-Liverpool-Wellcome Trust Clinical Research Programme, College of Medicine, University of Malawi, Blantyre, Malawi; 8 School of Public Health and Family Medicine, College of Medicine, University of Malawi, Blantyre, Malawi; 9 Faculty of Epidemiology and Population Health, London School of Hygiene and Tropical Medicine, London, UK

## Abstract

**Background:**

Malawi experienced prolonged use of sulfadoxine/pyrimethamine (SP) as the front-line anti-malarial drug, with early replacement of chloroquine and delayed introduction of artemisinin-based combination therapy. Extended use of SP, and its continued application in pregnancy is impacting the genomic variation of the *Plasmodium falciparum* population.

**Methods:**

Whole genome sequence data of *P. falciparum* isolates covering 2 years of transmission within Malawi, alongside global datasets, were used. More than 745,000 SNPs were identified, and differences in allele frequencies between countries assessed, as well as genetic regions under positive selection determined.

**Results:**

Positive selection signals were identified within *dhps*, *dhfr* and *gch1*, all components of the parasite folate pathway associated with SP resistance. Sitting predominantly on a *dhfr* triple mutation background, a novel copy number increase of ~twofold was identified in the *gch1* promoter. This copy number was almost fixed (96.8% frequency) in Malawi samples, but found at less than 45% frequency in other African populations, and distinct from a whole gene duplication previously reported in Southeast Asian parasites.

**Conclusions:**

SP resistance selection pressures have been retained in the Malawian population, with known resistance *dhfr* mutations at fixation, complemented by a novel *gch1* promoter duplication. The effects of the duplication on the fitness costs of SP variants and resistance need to be elucidated.

**Electronic supplementary material:**

The online version of this article (doi:10.1186/s12936-016-1634-6) contains supplementary material, which is available to authorized users.

## Background

Malawi suffers a heavy burden of endemic falciparum malaria with year-round transmission that peaks during the long rainy season from early December to May [1]. Malaria still accounts for 40% of hospitalizations in children under 5 years of age and 30% of all outpatient visits [2]. The malaria mortality rate is 63 per 100,000 population, and amongst the highest in East Africa [3] despite the roll out of control measures, such as insecticide-treated bed nets (ITNs), intensive indoor residual spraying (IRS), and artemisinin-based combination therapy (ACT) [2]. As one of the first African countries to switch from chloroquine to sulfadoxine/pyrimethamine (SP) in 1993, and the last to switch from SP to ACT in 2007, Malawi stands out from the rest of Africa in having a significantly prolonged exposure to SP [4]. Whilst this meant the reduced frequency of chloroquine resistance alleles in the *Plasmodium* population [5], the same cannot currently be said for SP resistance [6]. Resistance to SP is thought to be a cumulative process whereby mutations are successively acquired in both the *dhfr* (S108N, N51I, C59R, then I164L) and *dhps* (A437G then K540E) genes. These *dhps* and *dhfr* polymorphisms persist at high frequency in the Malawian *Plasmodium* population despite exposure to SP being reduced. Retention of these variants may be due in part to the use of SP in intermittent preventive treatment for pregnant women (IPTp) or a lower than expected fitness cost associated with these variants [6]. The scaling up of the distribution of ACT and IPTp contributed to a 36% drop in the mortality rate between 2004 and 2014 for children under 5 years of age, to an estimated 85 deaths per 1000 live births [2]. However, the control efforts could be derailed by the use of SP in IPTp strategies in sites where parasites resistant to SP persist, as well as any future emergence and spread of ACT-resistant parasites (as seen in Southeast Asia).

Genetic variation in *Plasmodium falciparum* is central to the parasite’s survival and can potentially undermine malaria control interventions. The evolutionary process enables parasite populations to select for variants that rapidly overcome host immune responses and anti-malarial drugs to establish persistent infections and increase transmission. Therefore, surveying evolutionarily driven genetic changes in *P. falciparum* and investigating parasite responses to anti-malarial interventions are crucial to efforts to reduce the malaria burden. Where regions are working towards pre-elimination, longitudinal samples are required to monitor parasite transmission dynamics and the efficacy of interventions to control malaria.

Previous work in a rural Malawian *P. falciparum* population in the Chikwawa district in a single ‘baseline’ season (n = 69, December 2010 to July 2011) identified several genes encoding merozoite invasion ligands as being retained in the population due to balancing selection [7]. This type of selection actively maintains multiple alleles in the gene pool of a population. Further, by comparing the Malawian *P. falciparum* population to others in Africa and Southeast Asia, signals of recent positive selection were identified at known drug targets (e.g., *dhps, crt* and *mdr1*), metabolic enzymes (e.g., *gch1*), and in several invasion ligands (e.g., *msp3.8, trap* and *ama1*) [7]. Initial analysis provided evidence of population divergence presumably driven by drug selection on *crt, dhps* and *mdr1* genes, and reflects the adaptation of parasite populations to local drug pressure, especially SP. The *dhps* (sulfadoxine target) and *dhfr* (pyrimethamine) genes are on the folate biosynthesis pathway of *P. falciparum*, and in Southeast Asian populations a copy number variant in *gch1* (first gene in pathway) is thought to be associated with SP resistance and its persistence [8].

The initial work in Malawi [7] was followed up by including additional ‘baseline’ season samples (n = 29, total n = 98) and comparing the genetic diversity in 122 isolates collected in the subsequent 2012 dry and wet seasons in the Chikwawa and Zomba districts, located 100 km apart. These regions are sentinel sites in Malawi, chosen for intensive anti-malarial intervention involving ACT, ITNs and IRS. The aim was to identify changes in allele frequency within individual, intra- and inter-season (wet and dry seasons) and identify regions under selection pressure. The findings demonstrate limited variation between the Malawian sub-populations over time and the impact of prolonged exposure of parasites to SP. Particularly, fixation of several known SP resistance SNPs and a novel copy number increase of the *gch1* promoter region were identified. Cross-population analysis revealed selective pressure for chloroquine resistance in non-Malawian populations. These populations have experienced prolonged use of chloroquine. Overall, the findings support the use of parasite and population genetic approaches to monitor transmission and the adaptation to drug pressure, and thereby inform the timing and type of interventions to be applied. Existing surveillance could be enhanced with rapid, field-based, genomic tests which genotype the *gch1* promoter region as a proxy for SP resistance in an African setting.

## Methods

### Study sites and sample collection

Whole blood samples were collected from October 2010 to November 2012 from children aged 5–28 months recruited in an ongoing ACTia[abbrev?] study within the high-transmission Chikwawa and Zomba regions in Malawi [7]. All individuals recruited had clinical falciparum malaria and received artemether/lumefantrine (AL) or dihydroartemisinin/piperaquine (DHA) treatment post-collection. Written informed consent was obtained from a parent or guardian of each child with the ethics committees of the University of Malawi’s College of Medicine and the Liverpool School of Tropical Medicine both approving the study.

### Whole-genome sequencing and quality control

Human DNA contamination was reduced through leukocyte-depletion of the blood samples using CF11 column filtration [9]. Purified DNA samples (n = 220) containing less than 30% human DNA were sequenced at the Sanger Institute using Illumina HiSeq2500 technology, with a minimum of 76-base, paired-end, fragment sizes. All short reads were mapped to the 3D7 reference genome (version 3.0) using *bwa*-*mem* [10]. SNPs and small indels were called using samtools and bcf/vcftools with default settings [10]. Only those variants with quality scores in excess of 30 (indicating an error rate less than one per 1000 bp) and with minimum coverage of ten were retained [10]. Genotypes at SNP positions were called using ratios of coverage and heterozygous calls were converted to the majority genotype on a 70:30 coverage ratio or greater [7, 11, 12]. SNPs were excluded from analysis if they had more than 5% mixed or missing genotype calls, or they were positioned within non-unique regions, sub-telomeric regions or within the hypervariable *var*, *rifin* and *stevor* gene families.

Raw sequencing data were also mapped for previously published *P. falciparum* strains (3D7, HB3, DD2, 7G8, GB4) [11] and isolates from East Africa (Kenya, Tanzania, n = 33), West Africa (Burkina Faso, The Gambia, Ghana, Guinea, Mali, Nigeria, n = 430), Central Africa [Democratic Republic of Congo (DRC), n = 56], South America (Colombia, Peru, n = 21), South Asia (Bangladesh, n = 54) and Southeast Asia (Cambodia, Laos, Myanmar, Papua New Guinea, Thailand, Vietnam, n = 1187) [7, 11–14] using the pipeline described above. Public accession numbers for raw sequence data analysed are contained in SRA studies ERP000190 and ERP000199, as well as being accessible from the Pf3k project website (https://www.malariagen.net/projects/pf3k). In total, the isolate dataset contains 745,913 high quality SNPs; 245,215 SNPs have no missing genotype calls, 77.1% within genes and 9.1% have a minor allele frequency greater than 1%.

### Statistical analysis

Population stratification in the isolates was investigated using a principal component analysis (PCA) of the pair-wise SNP distances between samples. This approach identified distinct African, Asian and South American clusters, with a further African-only investigation identifying West, Central and East African clusters. Differences in allele frequencies at each SNP were estimated using fixation indexes (F_ST_), with genes ranked by their maximum scores [15]. Tajima’s D [16] was implemented to identify genomic regions under balancing selection, with a score greater than two suggesting strong balancing selection. Scores were calculated for each gene containing at least four SNPs. All SNP-based analyses were performed using base R functions. Copy number variation in isolates and strains was analysed with DELLY using default settings [17].

Extended haplotype homozogysity (EHH)-based selection analyses, intra-population iHS and inter-population XP-EHH, were performed using selscan [18], using the default minor allele frequency and EHH truncation values of 0.05. Pair-wise country XP-EHH analyses used Malawi as the reference group, and both iHS and XP-EHH values were normalized genome-wide. P values for iHS and XP-EHH estimates were calculated using a Gaussian approximation. A significance threshold of P < 0.00006 was established for both iHS (>4) and XP-EHH (>6), using a simulation approach.

## Results

### Malawi sub-population analysis over time and location

Potential stratification within the Malawi dataset, across season, year and location of collection, was explored before consideration of shared signals. Of the 220 parasite isolates collected in this study, 85.9% were from the Chikwawa region whilst only 14.1% were from the Zomba region. Season-wise 43.2% were collected in a wet season, 56.8% in a dry season, across three years (2010 5.0%, 2011 39.5%, 2012 55.5%). Allele frequency differences between the location (median F_ST_ 0.003, max. 0.11) and year (median F_ST_ 0.004, max. 0.07) sub-populations were small. Within and between the seasons and location, the number of SNP differences was similar (~9400 SNPs) (see Additional file 1: Table S1). Twenty-six SNPs have an F_ST_ value greater than 0.15 for within-Malawi year, location and season-based sub-populations. Seven of these are intergenic and four within ‘conserved unknown’ genes (see Additional file 1: Table S2). The remaining 15 SNPs are within putative or known genes, including *surfin14.1* (max. F_ST_ 0.172), *heat shock protein 90* (max. F_ST_ 0.166) and the immune evasion antigen *PfEMP1* (max. F_ST_ 0.157) [19]. Notably, all top pair-wise F_ST_ values above 0.15 were from season-based comparisons. No major differences were detected in allele frequency for known drug resistance mutations (see Additional file 1: Table S3).

### Selection within the combined Malawian parasite sub-populations

**Fig. 1:**
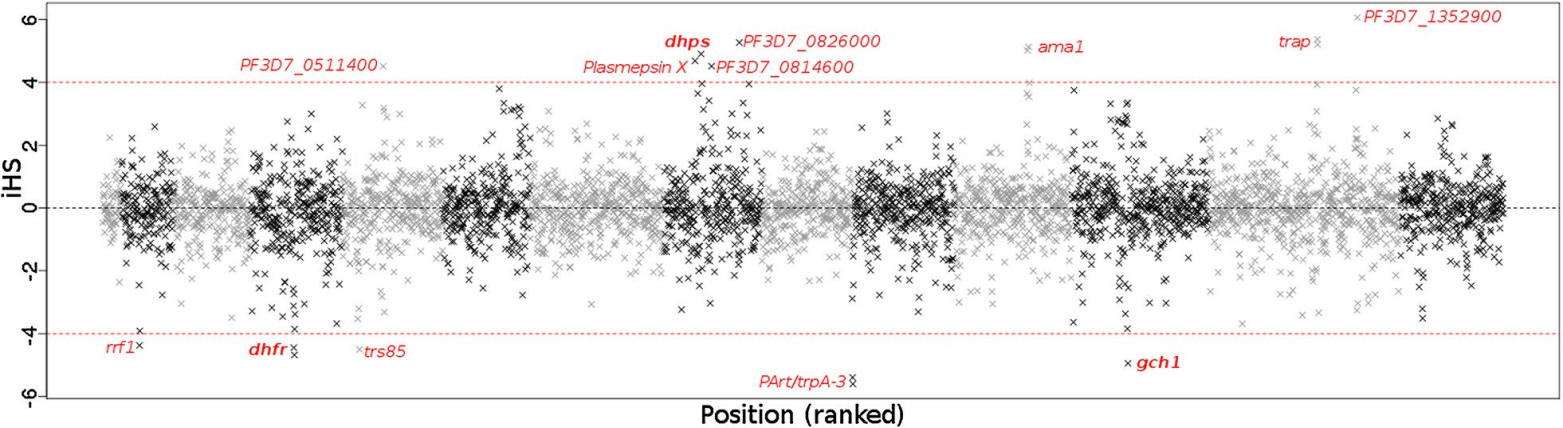
Whole genome integrated haplotype score (iHS) for the combined Malawian parasite population. *Red dotted lines* indicate threshold of normalized iHS scores greater than four

Given the absence of strong stratification between the sub-populations, the Malawian datasets were combined to identify selection signals within population. Signatures of potential recent positive selection were identified within 13 genes (iHS > 4). Five of these genes are currently uncharacterized, and three within close proximity to genes of known function and established selection sites (see Fig. 1; Additional file 1: Table S4). For example, the *PF3D7_1223400* signal (iHS: −4.948) is within 2 kbp of *gch1*, and loci were identified within 60 kbp of *dhps* and 8.5 kbp of *dhfr*, both members of the *P. falciparum* folate pathway [20]. Selection in these genomic regions within Malawi has been suggested previously [11] and relates to selective pressure due to use of SP.

Additional allele selection signals are present within the erythrocyte invasion critical protein *ama1* [21] (iHS: 5.129, 5.017) and the sporozoite surface protein *trap* (iHS: 5.366, 5.209), both representing hits that have been suggested previously [22, 23]. Non-reference allele selection loci were also found within *Plasmepsin X* (iHS: 4.686), which has a role in ookinete invasion [24]. In contrast, reference allele selection was identified centred on four loci within *TRS85* (iHS: −4.502), *RRF1* (iHS: −4.376) and *PArt/TrpA*-*3* (iHS: −5.604, −5.396). Three genes show potential balancing signals within the Malawian *P. falciparum* population (Tajima’s D > 2; see Additional file 1: Table S5). These are *ama1*, required for erythrocyte invasion [25], *surf8.2*, a group A SURFIN protein not found to be expressed post-invasion [26] but associated with susceptibility to pyrimethamine (inhibits folic acid metabolism via *dhfr*) [27], and a conserved unknown protein (*PF3D7_0710200*) within chromosome 7. The *ama1* locus has both balancing and positive selection, suggesting positive selection for one specific haplotype alongside the retention of multiple others. Such complex selection has previously been identified as differing between *ama1* domains [27, 28].

### Malawi within a global context

Parasite genetic diversity within Malawi was further contextualized within a global setting. A PCA approach identified distinct African, Asian and South American clusters, as previously reported using large SNP datasets [7, 11, 12, 14, 29]. Within the African-only analysis, distinct West, Central and East African clusters were also distinguished, and Malawi was separated from Tanzania and Kenya. (see Additional file 2: Figure S1).

Pair-wise F_ST_ values were calculated genome-wide for Malawi against each country with at least 14 samples. The median F_ST_ values per region per SNP were then estimated to identify those that contained Malawi-unique variants (see Table 1). The top hits included the *dhps* K540E causal mutation, likely reflecting the prevalence of SP resistance in Malawi and other East African populations considered, compared to elsewhere (Table 2; Additional file 1: Table S6). Of the remaining top hits, the study identified the *P47* and *P230* genes that share roles in gametocyte fertility [30], P48/45 reflecting differences in mosquito vectors [30], and *PF3D7_0913900* a putative arginine-tRNA ligase. When considering drug-resistant candidate polymorphism, *crt* mutations (K76T, Q271E, N326S, I356T) were absent in Malawi, reflecting early withdrawal of chloroquine compared to other African countries (see Table 2; Additional file 1: Table S6). K76T and Q271E mutations are near fixed in Asia, and known to have undergone ‘hard’ selective sweeps [31]. Compared to other African populations, Malawi has a higher frequency of *dhfr* triple mutant haplotypes (N51I/C59R/S108N) and *dhfr⁄dhps* quintuple mutant genotypes (*dhfr* N51I/C59R/S108N haplotype and *dhps* A437G/K540E haplotype). The *dhps* S436A mutation was at high frequency in West Africa, and almost absent in Malawi. The contributing *dhfr* quadruple mutation (I164L) and *dhfr⁄dhps* sextuple mutant genotypes (*dhfr⁄dhps* quintuple mutant genotype and *dhfr* I164L) were only present in Asia. In Malawi, the *dhps* A581G mutation, which has been shown to reduce the effectiveness of SP preventive therapy [32] was present at low frequency, leading to the presence of an alternative *dhfr⁄dhps* sextuple genotype (*dhfr⁄dhps* quintuple genotype and *dhfr* I164L). In the Malawian population, no variants of the *kelch13* gene, previously described in Southeast Asia to be associated with artemisinin resistance [33], were identified. All alternative alleles in *kelch13* are present at low frequencies, the maximum at 0.091 for K189T, and reflect previous SNPs frequencies for the rest of Africa [34].

**Table 1:** Differences in allele frequencies between Malawi and other populations (based on pairwise F_ST_ scores)

Gene ID	Position	Gene	Other East Africa	DRC	West Africa	South Asia	Southeast Asia	South America
*PF3D7_0810800*	549,993	*dhps*	0.053	*0.873*	*0.974*	0.118	0.415	*0.628*
*PF3D7_0209000*	375,427	*P230*	0.023	0.035	*0.689*	*0.722*	*0.569*	*0.775*
*PF3D7_1016500*	663,199	*PHISTc*	0.023	0.077	*0.504*	0.463	0.174	0.775
*PF3D7_0913900*	596,674	Arginine-tRNA ligase	0.017	0.014	*0.769*	*0.665*	*0.719*	*0.518*
*PF3D7_1339700*	1,595,988	Conserved unknown	0.013	0.017	0.323	*0.555*	*0.731*	0.213
*PF3D7_1032100*	1,293,621	*dcp1*	0.021	0.132	*0.709*	*0.578*	*0.659*	0.485
*PF3D7_1223500*	958,593	Conserved unknown (near *gch1*)	0.093	0.285	*0.535*	*0.668*	*0.667*	*0.668*
*PF3D7_1361800*	2,481,275	Conserved unknown	0.02	0.019	*0.533*	*0.630*	*0.606*	*0.665*
*PF3D7_0708500*	385,921	*hsp86*	0.292	0.297	0.428	*0.542*	*0.625*	0.016
*PF3D7_1346800*	1,880,114	*P47*	0.055	0.26	0.434	*0.590*	*0.590*	*0.590*
*PF3D7_0113000*	489,337	*garp*	0.024	0.064	*0.562*	*0.587*	0.478	0.104
*PF3D7_0716700*	730,051	Conserved unknown	0.034	0.079	0.366	*0.533*	*0.567*	*0.567*
*PF3D7_0307900*	350,293	Conserved unknown	0.016	0.169	*0.524*	0.267	0.487	*0.524*
*PF3D7_1248700*	1,997,660	Conserved unknown	0.131	0.211	0.457	0.426	0.457	0.459
*PF3D7_1223400*	942,564	Phospholipid-transporting ATPase (near *gch1*)	0.172	0.237	0.380	0.444	0.444	0.444
*PF3D7_1223300*	938,341	*gyrA* (near *gch1*)	0.146	0.237	0.374	0.444	0.308	0.444
*PF3D7_1223400*	941,821	Phospholipid-transporting ATPase (near *gch1*)	0.171	0.216	0.381	0.442	0.442	0.442
*PF3D7_1426700*	1,036,865	*pepc*	0.022	0.001	0.429	0.251	0.419	0.430
*PF3D7_0215300*	629,060	*acs8*	0.174	0.104	0.402	0.393	0.426	0.132
*PF3D7_1346700*	1,876,606	*P48/45*	0.120	0.042	0.324	0.426	0.402	0.220
*PF3D7_0615900*	665,589	Conserved unknown	0.002	0.261	0.37	0.311	0.343	0.373
*PF3D7_1433900*	1,362,042	Putative protein kinase	0.051	0.012	0.233	0.368	0.340	0.368
*PF3D7_0811600*	586,054	Conserved unknown	0.003	0.065	0.340	0.358	0.304	0.358
*PF3D7_0724900*	1,056,801	Putative kinesin-19	0.010	0.099	0.220	0.358	0.344	0.358
*PF3D7_1414200*	564,437	Conserved unknown	0.003	0.083	0.278	0.333	0.333	0.333
*PF3D7_1021700*	883,159	Conserved unknown	0.027	0.201	0.317	0.300	0.331	0.331
*PF3D7_0627800*	1,115,191	Putative acetyl-CoA synthetase	0.042	0.325	0.325	0.325	0.325	0.325
*PF3D7_1218300*	718,254	*Ap2mu*	0.029	0.312	0.309	0.312	0.312	0.312
*PF3D7_1223100*	928,407	PKAr	0.231	0.306	0.306	0.306	0.306	0.306
*PF3D7_1222600*	911,963	*AP2*-*G*	0.218	0.274	0.304	0.304	0.297	0.304
*PF3D7_1223300*	935,411	*gyrA* (near *gch1*)	0.213	0.306	0.306	0.306	0.306	0.306
*PF3D7_0932800*	1,306,240	Conserved unknown	0.063	0.274	0.304	0.304	0.297	0.304

Values are the median F_ST_ values for each regional population. In italics are regional medians above 0.5

**Table 2:** Drug resistance allele frequencies

SNP	Malawi	Other East Africa	DRC	West Africa	South Asia	South East Asia	South America
Sample size	220	33	56	430	54	1133	21
*dhps*							
S436A	0.005	0.061	0.107	0.505	0.509	0.313	0
A437G	0.998	0.894	0.902	0.735	0.843	0.971	0.286
K540E	0.995	0.894	0.063	0.014	0.778	0.477	0.190
A581G	0.027	0.091	0.027	0.002	0.156	0.407	0.238
*dhfr*							
N51I	0.991	0.909	0.982	0.658	0.471	0.897	0.381
C59R	0.991	0.939	0.821	0.745	0.972	0.996	0
S108N	1	1	1	0.781	1	0.998	0.952
I164L	0	0	0	0	0.167	0.438	0
*Double dhfr mutant* ^a^	1	1	1	0.728	0.982	0.995	0.381
*Triple dhfr mutant* ^b^	0.977	0.848	0.750	0.563	0.392	0.887	0
*Quadruple dhfr Mutant* ^c^	0	0	0	0	0.167	0.438	0
*dhfr*-*dhps*							
*Quintuple genotype* ^d^	0.968	0.788	0.036	0.007	0.278	0.421	0
*Quintuple* + *dhfr I164L*	0	0	0	0	0.130	0.266	0
*Quintuple* + *dhps A581G*	0.027	0.091	0.027	0	0.111	0.198	0
*crt*							
K76T	0	0.485	0.661	0.416	0.889	0.960	1.000
Q271E	0	0.485	0.643	0.430	0.907	0.935	0
N326S	0	0	0	0.002	0.241	0.630	0
I356T	0	0	0.196	0.140	0.833	0.652	0
*kelch13*							
K189T	0.091	0.061	0.196	0.502	0.130	0.007	0.714
K189 N	0.005	0	0	0.026	0	0	0
Y493H	0	0	0	0	0	0.044	0
C580Y	0	0	0	0	0	0.224	0
*gch1*							
Promoter duplication	0.968	0	0.446	0.251	0.037	0.003	0
Whole gene duplication	0	0	0.020	0.023	0.111	0.076	0

*DRC* Democratic Republic of Congo

^a^Any two of N51I, C59R or S108N

^b^N51I, C59R & S108N

^c^Triple *dhfr* mutant haplotype with I164L

^d^ *dhfr* N51I/C59R/S108N haplotype + *dhps* A437G/K540E haplotype

The XP-EHH method was used to identify regions under selection in the Malawi population compared to others. Positive values suggest relative selection in Malawi, whilst negative values suggest selection in non-Malawi (see Additional file 2: Figure S2; Additional file 1: Table S7). 31 genes (|XP-EHH| > 6) were identified, of which 18 are uncharacterized and 14 appear against only one other country. The most striking signals were within *PF3D7_1223400* and *PF3D7_1223500*, both uncharacterized but within 2 kbp of *gch1*, and indicate relative selection within the Malawian population when compared to Burkina Faso, the DRC, The Gambia, Ghana, Guinea, Kenya, and Mali (see Additional file 1: Table S7; Additional file 2: Figure S2). Strong shared negative XP-EHH scores were also present within *acs8*, the uncharacterized *PF3D7_1421100* and the SP resistance gene *dhps.* Strong positive signals, suggestive of selection in the non-Malawian populations, were present within *msp10*, *trap* and three genes directly downstream of *crt* (*cg1*, *glp3*, *cg2)*.

### Adaptive copy number selection in the *gch1* promoter

**Fig. 2:**
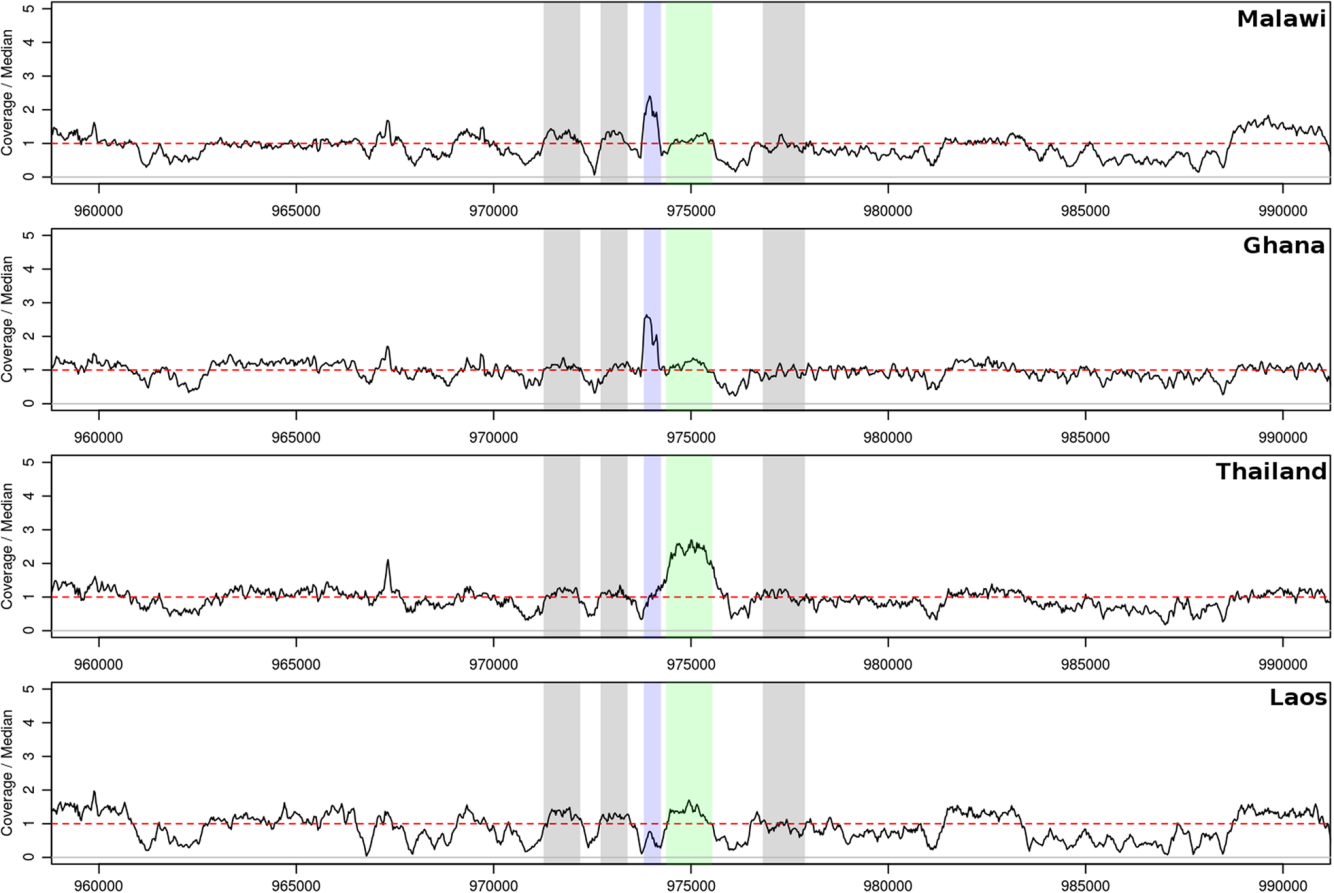
Coverage plots displaying *gch1* whole gene and promoter duplications. The Malawi (ERR237436) and Ghanaian (ERR045593) samples represent promoter duplications (upstream of the *gch1* coding region) of approximately twofold, the Thailand sample (ERR248939) represents whole gene duplication (*green* region) and the Laos sample (ERR216496) represents no duplication. Neighbouring non-*gch1* genes are displayed as the *grey* regions. Coverage is scaled against the median coverage (indicated by the *dashed red line*) for chromosome 12 and calculated for 100 bp windows, with an offset of 25 bp

**Fig. 3:**
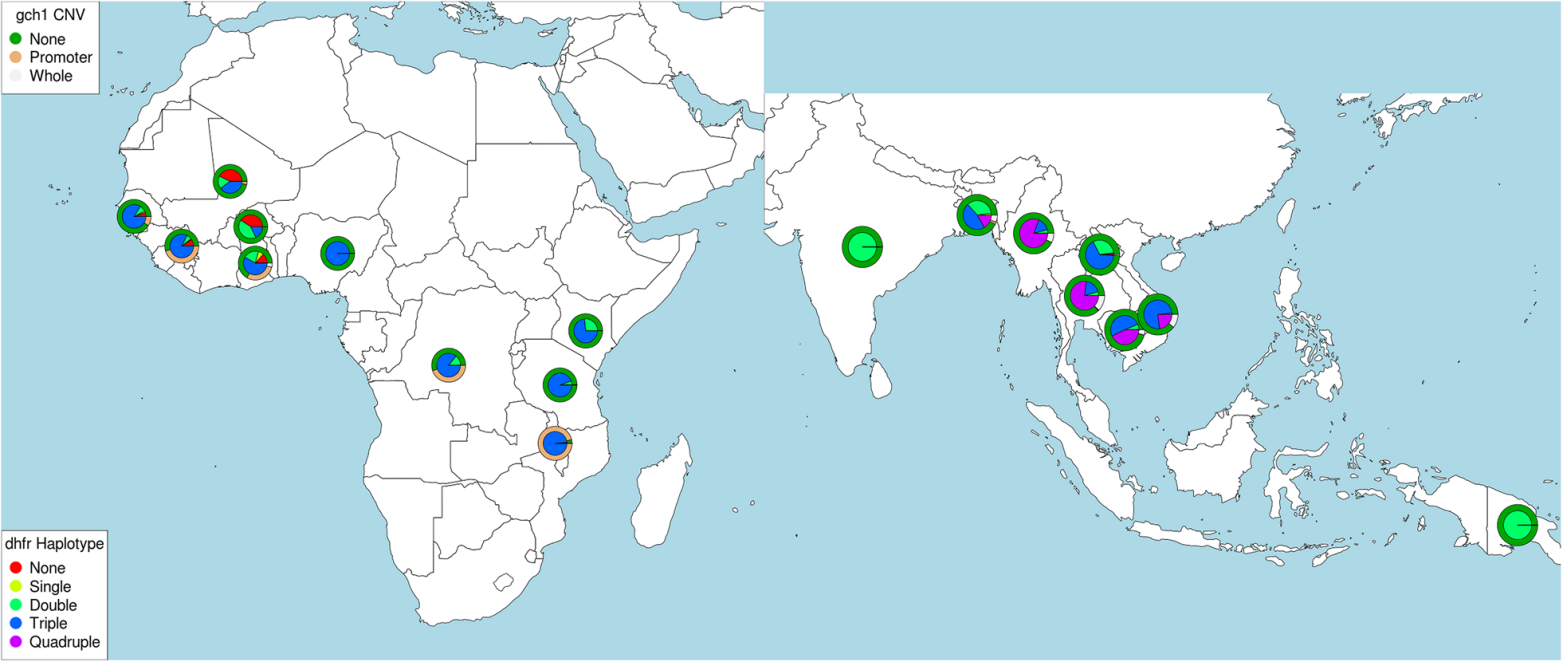
Relationship between *dfhr* mutations and *gch1* copy numbers. *Plasmodium falciparum* parasites with DELLY-predicted *gch1* promoter duplication across Africa exist almost exclusively on a *dhfr* triple mutant background, whilst predicted whole gene duplication in South East Asia is linked to the *dhfr ‘*quadruple mutant’

Given the hypothesized role for copy number variation (CNV) in *gch1*-mediated SP resistance [35] and signals of selection observed, potential CNVs at this locus were investigated. Evidence of a promoter duplication was found (genomic region 973,804–974,240 bp; see Fig. 2) in almost all Malawian samples (n = 213, 96.8%; see Table 2; Additional file 1: Table S6). Similar promoter duplications were found in samples from Ghana (n = 59, 29.2%), Guinea (n = 43, 45.3%), DRC (n = 25, 44.6%), The Gambia (n = 5, 9.1%), and Asia (n = 5, < 0.4%). In contrast, the whole *gch1* gene duplication [8] was predicted in samples from Thailand (n = 29, 13.8%), Cambodia (n = 23, 4.4%), Vietnam (n = 20, 10.7%), Ghana (n = 10, 5%), Myanmar (n = 7, 7.4%), and Bangladesh (n = 6, 11.1%) (see Fig. 2; Table 2; Additional file 1: Table S6). The gene duplication appears predominantly on the background of *dhfr* triple mutant haplotype in West Africa (79.2%) and a quadruple mutant haplotype in Southeast Asia (80.3%) and South Asia (50.0%) (see Table 3). The promoter duplication is seemingly not linked to the *dhfr* I164L quadruple mutation and sits predominantly on a triple mutant haplotype background in Malawi (99.1%), DRC (87.9%) and West Africa (85.8%) (see Fig. 3). In these populations, the haplotype background of *dhps* S436A, A437G and K540E appears less important with a number of mutation combinations present (Table 3). The exception is Malawi where the promoter sits on a predominantly *dhps* A437G/K540E haplotype background, leading to the high frequency of quintuple mutant genotypes.

**Table 3:** Co-association frequencies of *dhfr* mutations and *gch1* duplications

*dhfr* mutants^a^	*gch1* duplication	*dhps* mutants^b^	Malawi	Other East Africa	DRC	West Africa	South Asia	Southeast Asia	South America
None	None	None	0	0	0	0.067	0	0.002	0.048
None	None	Single	0	0	0	0.095	0	0	0
Single	None	None	0	0	0	0.002	0.019	0.002	*0.524*
Single	None	Single	0	0	0	0.040	0	0	0.048
Double	None	None	0	0.030	0.018	0.030	0.019	0.031	0.143
Double	None	Single	0	0	0.071	0.112	0.019	0.028	0
Double	None	Double	0.005	0.121	0	0.002	*0.278*	0.021	0.143
Double	Promoter	None	0	0	0	0.002	0	0	0
Double	Promoter	Single	0	0	0.054	0.028	0	0	0
Double	Promoter	Double	0.009	0	0	0	0	0	0
Double	Gene	Single	0	0	0	0.005	0	0	0
Double	Gene	Double	0	0	0	0	0.037	0.001	0
Triple	None	None	0	0.061	0.036	0.086	0.019	0.078	0
Triple	None	Single	0	0	*0.393*	*0.328*	0.056	0.190	0.048
Triple	None	Double	0.032	*0.788*	0.036	0.005	*0.389*	0.185	0.048
Triple	Promoter	None	0	0	0.036	0.012	0	0	0
Triple	Promoter	Single	0	0	*0.304*	0.160	0	0	0
Triple	Promoter	Double	*0.950*	0	0.054	0.009	0	0	0
Triple	Gene	Single	0	0	0	0.014	0	0.007	0
Triple	Gene	Double	0	0	0	0	0	0.006	0
Quadruple	None	None	0	0	0	0	0.019	0.004	0
Quadruple	None	Single	0	0	0	0	0.019	0.144	0
Quadruple	None	Double	0	0	0	0	0.093	*0.244*	0
Quadruple	Gene	Single	0	0	0	0	0	0.014	0
Quadruple	Gene	Double	0	0	0	0	0.037	0.042	0

Frequencies greater than 0.2 are shown in italics

^a^ *dhfr* mutant haplotypes: double—any two of *dhfr* N51I, C59R or S108N; triple—*dhfr* N51I, C59R and S108N; quadruple—*dhfr* triple mutant haplotype with I164L; DRC Democratic Republic of Congo

^b^ *dhps* double mutant haplotypes consist of *dhps* A437G and K540E

**Fig. 4:**
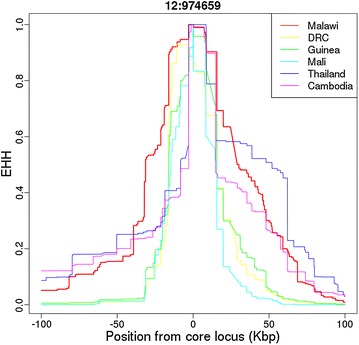
EHH plots for multiple populations, centred on a SNP within *gch1* (chromosome 12, position 974,659)

Coverage analysis of the *P. falciparum* reference strains 3D7, HB3, DD2, 7G8, and GB4 identified no upstream promoter duplication. Previously identified whole gene duplications in 3D7, 7G8, DD2, and GB4, and the absence of duplication in HB3, were confirmed [36] (see Additional file 2: Figure S4). 38 SNPs (eight non-synonymous) were identified across the *gch1* coding region using the global dataset, all with non-reference allele frequencies (<4%), except a SNP in South America (position 974,633, 28.3%). In the *gch1* promoter region there were 11 SNPs occurring only in African populations, all at low frequency (<4%), except for one polymorphism, which was almost always found on a non-duplication background (position 974,046: East Africa 10.0%, DRC 18.3%, West Africa 18.5%). The overall low levels of nucleotide variation and sequence homogeneity support the argument that *gch1* copy number variants, rather than associated coding SNPs, are targeted by selection. Examination of the EHH revealed differences in linkage disequilibrium (LD) between continents and within Africa (see Fig. 4; Additional file 2: Figure S2). When considering African populations, there is evidence in non-Malawian populations of near symmetrical decays of EHH to a level close to zero within 25 kb. Malawi LD extends much wider and is consistent with a sweep around the promoter duplication (see Fig. 4).

To determine whether the *gch1* promoter duplication was under relative positive selection in the DRC, Ghana and Guinea, a sub-population was applied XP-EHH analysis, where those with the duplication were compared to those without. This approach identified no significant differential sub-population selection in either the DRC, Ghana or Guinea (|XP-EHH| < 4), although there are near-significant positive selection signals for the duplication-positive sub-populations in Ghana and Guinea (see Additional file 2: Figure S3). This contrasts with Malawi, where the duplication appears to be under active selection, potentially reflecting differences in SP usage (see Fig. 1; Additional file 2: Figure S2).

## Discussion

Malaria surveillance is crucial to informing and supporting disease prevention, control and elimination strategies. Access to technological advances and their reduced costs mean monitoring approaches involving genomic data are being implemented within malaria programmes [7, 12, 14]. To inform such programmes, genomic differences were investigated in Malawian *P. falciparum* parasites from two regions across multiple seasons. No major differences were observed between the two regional or temporal sub-populations, potentially due to small sample sizes, a narrow two-year study period, the removal of a minority of SNPs with a high frequency of missing or heterozygous genotypes, and study regions that are only 100 km apart with high human migration between them.

Because of the effects of control measures on *Plasmodium* genomes, signals of selection and differences in allele frequencies were also investigated across Malawi parasites and compared with those from global datasets. Signals in drug resistance-associated genes, and surface-associated proteins that are exposed to the host immune system were detected, including previously described selection in *msp10*, *trap* and *ama1* [7, 23]. Antibodies against the pre-erythrocyte TRAP and erythrocyte stages MSP10 and AMA1 proteins have been detected in anti-malarial-acquired immunity individuals living in malaria-endemic areas [37, 38]. Vaccines using TRAP, AMA1 and MSP proteins (e.g., MSP1 and MSP3) are being tested with promising results [39]; as such the MSP10 protein may represent a viable vaccine candidate.

Consistent with previous reports, *crt* mutations (K76T, Q271E, N326S, I356T) were absent from Malawi [40, 41]. Neighbouring countries (as well as others in Africa) that did not switch away from chloroquine as early as Malawi continue to report higher levels of *crt* mutations. This observation has led to a setting likened to a Malawian island of chloroquine drug sensitivity in a sea of resistance [41]. Three genes, *cg1, glp3* and *cg6*, immediately downstream of the *crt* gene, displayed high relative positive selection in multiple non-Malawian populations, consistent with hard selective sweeps [31]. In contrast, SP resistance-associated *dhfr* and *dhps* alleles are retained at high frequencies in Malawi. Compared to other African populations there is a significantly higher level of both the *dhfr* C59R mutation and the quintuple *dhfr⁄dhps* mutant genotypes. These frequencies are consistent with previous analyses highlighting the pan-African distribution of the *dhfr* triple mutant haplotypes in contrast to the East–West African differences in distribution of the A437G and K540E *dhps* variants [42, 43]. The SNP-based work is consistent with the results from microsatellite-based studies, considering the dynamics of strong selection for mutations conferring SP resistance, including support for the observation of the independent origin of sulfadoxine-resistant alleles across a number of regions [42, 44, 45]. Together, these observations reflect the early decline in chloroquine usage and early introduction and prolonged use of SP in Malawi when compared to other African populations.

In almost all Malawian samples (96.8%), a novel duplication was identified in the promoter of the *gch1* gene (973,804–974,240 bp), a member of the folate pathway that includes targets for sulfadoxine and pyrimethamine. This duplication was also detected in other parasites, particularly from West Africa and the DRC, but is near-absent in Asia. Whole gene duplications have previously been detected in Thailand and Cambodia [33] and were detected in this dataset. Positive selection signals are present across the *gch1 region* in Malawi, with relative selection in Malawi compared to other African populations where the promoter duplication is also present. Further, samples with the duplication were not found to be under relative selection in the DRC, Ghana or Guinea. Together, the strong evidence of selection for this promoter duplication in Malawi supports its role as being advantageous for *P. falciparum* parasites in regions with high SP use, particularly where there is a higher frequency of resistance-associated *dhfr* and possibly *dhps* variants.

The function of the promoter duplication remains to be established. It is possible that both promoter and whole gene duplications increase *gch1* expression in vivo thereby reducing the fitness cost associated with *dhfr* and *dhps* variants, which convey resistance to SP. *In silico,* functional predictions for the promoter duplication identified multiple TATA, TATAA and TGTAA PfTBP binding motifs [46]. If this duplication acts to reduce the fitness cost of other SP resistance-associated variants, its presence may suggest a more persistent form of resistance which further surveillance will need to confirm. Ongoing fieldwork in Malawi will allow to survey the parasite population during longer periods and to detect genomic changes following the introduction of ACT. Further work should also consider the genomic landscape within other African countries to determine the frequency of the *gch1* duplication and other variants associated with SP resistance.

## Conclusion

This study reports the persistence of genetic variants associated with SP and chloroquine resistance within the *P. falciparum* population in Malawi, despite withdrawal of these anti-malarials from front-line use. Signals of positive selection were also identified, which suggest retention of these resistance-associated variants, as well as various life stage-specific surface antigens. Investigation of *gch1* copy number variation identified the near fixation of a specific 436 bp promoter duplication within Malawi, present in other African countries but absent from Asian populations. It is most likely that this promoter duplication acts in a similar fashion to the whole gene duplication present in Asian populations, although further experimental work is required to elucidate any functional impact.

## Additional files

**Additional file 1.** Additional tables.

**Additional file 2.** Additional figures.
