# Supplementary material for: Characterizing the impact of sustained sulfadoxine/pyrimethamine use upon the Plasmodium falciparum population in Malawi
Source: Malar J. 2016 Nov 29;15:575. doi: 10.1186/s12936-016-1634-6 (PMC5129638; doi:10.1186/s12936-016-1634-6)
Supplement: Supplementary file 1 — Additional file 1. Additional tables. [file 12936_2016_1634_MOESM1_ESM.docx]

**Additional Table 1:** Pairwise differences between Malawi seasonal sub-populations.

|  | **Chikwawa**  **2011 (Wet)**  **(n=22)** | **Chikwawa**  **2011 (Dry)**  **(n=64)** | **Chikwawa**  **2012 (Wet)**  **(n=34)** | **Chikwawa**  **2012 (Dry)**  **(n=57)** | **Zomba**  **2012 (Wet)**  **(n=31)** |
| --- | --- | --- | --- | --- | --- |
| **Chikwawa**  **2011 (Wet)**  **(n=22)** | 9458  (7607 -10400) | 9434  (6130-11020) | 9458  (6229-10660) | 9538  (5951-10640) | 9522  (6131-10740) |
| **Chikwawa**  **2011 (Dry)**  **(n=64)** | 0.01  (0.00-0.19) | 9381  (4560 - 11190) | 9395  (4484-11140) | 9474  (4270-11100) | 9442  (4681-11140) |
| **Chikwawa**  **2012 (Wet)**  **(n=34)** | 0.01  (0.00-0.20) | 0.01  0.00-0.12 | 9396  (900-10710) | 9485  (4440-10810) | 9438  (4724-10770) |
| **Chikwawa**  **2012 (Dry)**  **(n=57)** | 0.01  (0.00-0.19) | 0.01  (0.00-0.12) | 0.01  (0.00-0.12) | 9557  (4758 - 10700) | 9536  (4651-10880) |
| **Zomba**  **2012 (Wet)**  **(n=31)** | 0.01  (0.00-0.22) | 0.01  (0.00-0.19) | 0.01  (0.00-0.15) | 0.01  (0.00-0.14) | 9448  (522 - 10680) |

Upper and diagonal values indicate median (range) number of SNP differences between individuals. Lower values indicate median (range) F_ST_ values across SNPs for each sub-population pair.

**Additional** **Table 2:** Top F_ST_ values for pairwise Malawi sub-population comparisons.

| **Locus** | **Gene** | **Max Fst** | **Other Fst** |
| --- | --- | --- | --- |
| *PF3D7_1038400* | Gametocyte specific protein | 0.220 | 0.001 – 0.202 |
| *PF3D7_1008500* | Conserved unknown | 0.199 | 0.000 – 0.187 |
| *PF3D7_0420000* | Putative zinc finger | 0.198 | 0.001 – 0.152 |
| 8:1309330 | Intergenic | 0.191 | 0.001 – 0.097 |
| 7:1345508 | Intergenic | 0.188 | 0.000 – 0.128 |
| *PF3D7_0707300* | Rhoptry-associated membrane antigen | 0.180 | 0.002 – 0.118 |
| *PF3D7_1326400* | Putative translation initiation factor eIF-2B | 0.178 | 0.000 – 0.126 |
| 4:1061702 | Intergenic | 0.178 | 0.000 – 0.172 |
| *PF3D7_0710000* | Conserved unknown | 0.174 | 0.000 – 0.050 |
| *PF3D7_0525000* | Putative zinc finger | 0.173 | 0.009 – 0.118 |
| *PF3D7_1446500* | Conserved unknown | 0.173 | 0.001 – 0.134 |
| *PF3D7_1477600* | SURFIN 14.1 | 0.172 | 0.000 – 0.117 |
| 4:545378 | Intergenic | 0.170 | 0.001 – 0.071 |
| *PF3D7_0708400* | Heat Shock Protein 90 | 0.166 | 0.001 – 0.142 |
| *PF3D7_0709300* | Putative Cg2 protein | 0.165 | 0.005 – 0.084 |
| 6:824765 | Intergenic | 0.164 | 0.001 – 0.132 |
| 8:698268 | Intergenic | 0.162 | 0.001 – 0.083 |
| *PF3D7_1200700* | Acyl-CoA Synthetase | 0.158 | 0.000 – 0.121 |
| *PF3D7_1135600* | Putative condensin-2 complex subunit D3 | 0.158 | 0.000 – 0.053 |
| 9:91336 | Intergenic | 0.157 | 0.000 – 0.144 |
| *PF3D7_0905400* | High molecular weight rhoptry protein 3 | 0.157 | 0.002 – 0.078 |
| *PF3D7_0412400* | PfEMP1 | 0.157 | 0.000 – 0.076 |
| *PF3D7_0310200* | Putative phd finger protein | 0.156 | 0.001 – 0.132 |
| *PF3D7_1325400* | Conserved unknown | 0.152 | 0.001 – 0.105 |
| *PF3D7_0629300* | Putative phospholipase | 0.151 | 0.001 – 0.128 |

Threshold of greater than 0.15.

**Additional Table 3:** Allele frequencies for Sulfadoxine-Pyrimethamine resistance mutations within Malawi across all seasons.

| **Genetic variant** | **Chikwawa**  **2011 Wet** | **Chikwawa**  **2011 Dry** | **Chikwawa**  **2012 Wet** | **Chikwawa**  **2012 Dry** | **Zomba**  **2012 Wet** | **Combined population** |
| --- | --- | --- | --- | --- | --- | --- |
| Sample Size | 22 | 64 | 38 | 57 | 27 | 220 |
| *dhps* |  |  |  |  |  |  |
| S436A | 0 | 0 | 0.026 | 0 | 0 | 0.005 |
| A437G | 1 | 1 | 0.987 | 1 | 1 | 0.998 |
| K540E | 1 | 0.992 | 0.987 | 1 | 1 | 0.995 |
| *dhfr** |  |  |  |  |  |  |
| N51I | 1 | 1 | 1 | 0.983 | 1 | 0.991 |
| C59R | 0.955 | 0.984 | 1 | 0.991 | 1 | 0.991 |
| S108N | 1 | 1 | 1 | 1 | 1 | 1 |
| *Triple mutant*** | 0.955 | 0.984 | 1 | 0.965 | 1 | 0.977 |
| *gch1**** |  |  |  |  |  |  |
| Promoter duplication | 1 | 0.938 | 0.974 | 0.965 | 1 | 0.968 |

*I164L (quadruple mutation) is not present; ** *dhfr* N51I, C59R & S108N haplotype; *** Whole gene duplication of *gch1* is absent.

**Additional Table 4:** Top hits for Malawi-only positive selection (iHS) analysis.

| **Gene ID** | **Position** | **iHS** | **Gene** |
| --- | --- | --- | --- |
| *PF3D7_0208600* | 355154 | -4.376 | *RRF1* |
| *PF3D7_0417400* | 758290, 758269 | -4.678, -4.448 | Conserved unknown **(near *dhfr)*** |
| *PF3D7_0505100* | 226777 | -4.502 | *TRS85* |
| *PF3D7_0511400* | 481921 | 4.516 | Conserved unknown |
| *PF3D7_0808200* | 417751 | 4.686 | *Plasmepsin X* |
| *PF3D7_0809600* | 484954, 490762 | 4.906, 3.962 | Putative petidase family C50 **(near *dhps*)** |
| *PF3D7_0814600* | 703454 | 4.519 | Conserved unknown |
| *PF3D7_0826000* | 1111727 | 5.272 | Conserved unknown |
| *PF3D7_1002200* | 1115470, 1115617 | -5.604, -5.396 | *PArt/TrpA-3* |
| *PF3D7_1133400* | 1294082, 1294982 | 5.129, 5.017 | *ama1* |
| *PF3D7_1223400* | 1943339 | -4.947 | Phospholipid-transporting ATPase **(near *gch1*)** |
| *PF3D7_1335900* | 1466252, 1466264 | 5.366, 5.209 | *trap* |
| *PF3D7_1352900* | 2114996 | 6.066 | Exported unknown |

Positive scores indicate selection for the alternative core allele, whilst negative scores indicate selection for the reference core allele. All hits are above a threshold of 4 ( |iHS|>4 ).

**Additional Table 5:** Top Tajima’s D values for the combined Malawi population.

| **Gene ID** | **Gene** | **Tajima’s D** |
| --- | --- | --- |
| *PF3D7_0710200* | Conserved unknown | 3.224 |
| *PF3D7_0830800* | *surf8.2* | 3.196 |
| *PF3D7_1133400* | *ama1* | 2.916 |
| *PF3D7_0424400* | *surf4.2* | 1.605 |
| *PF3D7_1335900* | *trap* | 1.503 |
| *PF3D7_0113800* | DBL-containing protein | 1.475 |
| *PF3D7_1475900* | Conserved unknown | 1.205 |
| *PF3D7_1004800* | Putative ADP/ATP carrier protein | 1.080 |
| *PF3D7_1035700* | Duffy binding-like merozoite surface protein | 0.970 |

**Additional Table 6:** Drug resistance candidate mutation frequencies

| **Gene/SNP** | **Malawi** | **Tanzania** | **Kenya** | **DRC** | **Burkina Faso** | **Gambia** | **Ghana** | **Guinea** | **Mali** | **Nigeria** | **Bangladesh** | **Cambodia** | **Myanmar** | **Papua New Guinea** | **Laos** | **Thailand** | **Vietnam** | **Colombia** | **Peru** |
| --- | --- | --- | --- | --- | --- | --- | --- | --- | --- | --- | --- | --- | --- | --- | --- | --- | --- | --- | --- |
| Sample Size | 220 | 18 | 15 | 56 | 39 | 55 | 202 | 95 | 35 | 4 | 54 | 527 | 95 | 11 | 104 | 210 | 187 | 14 | 7 |
| *dhps* |  |  |  |  |  |  |  |  |  |  |  |  |  |  |  |  |  |  |  |
| S436A | 0.005 | 0.056 | 0.067 | 0.107 | 0.538 | 0.100 | 0.609 | 0.458 | 0.657 | 0.250 | 0.509 | 0.342 | 0.332 | 0 | 0.212 | 0.250 | 0.366 | 0 | 0 |
| A437G | 0.998 | 0.944 | 0.833 | 0.902 | 0.615 | 0.764 | 0.705 | 0.705 | 0.257 | 1.000 | 0.84 | 0.895 | 1.000 | 0.227 | 0.572 | 1.000 | 0.799 | 0.071 | 0.714 |
| K540E | 0.995 | 0.944 | 0.833 | 0.062 | 0 | 0 | 0.010 | 0.042 | 0 | 0 | 0.778 | 0.341 | 0.9 | 0.136 | 0.178 | 0.907 | 0.348 | 0 | 0.571 |
| *dhfr* |  |  |  |  |  |  |  |  |  |  |  |  |  |  |  |  |  |  |  |
| N51I | 0.991 | 0.944 | 0.867 | 0.982 | 0.359 | 0.918 | 0.592 | 0.821 | 0.486 | 1.000 | 0.471 | 0.925 | 0.901 | 0 | 0.644 | 0.938 | 0.963 | 0.214 | 0.714 |
| C59R | 0.991 | 1.000 | 0.867 | 0.821 | 0.423 | 0.845 | 0.757 | 0.879 | 0.486 | 1.000 | 0.972 | 0.996 | 1.000 | 0.955 | 0.976 | 1.000 | 1.000 | 0 | 0 |
| S108N | 1.000 | 1.000 | 1.000 | 1.000 | 0.397 | 0.936 | 0.817 | 0.879 | 0.471 | 1.000 | 1.000 | 1.000 | 1.000 | 1.000 | 0.976 | 1.000 | 1.000 | 0.929 | 1.000 |
| I164L | 0 | 0 | 0.052 | 0 | 0 | 0 | 0 | 0 | 0 | 0 | 0.382 | 0.432 | 0.861 | 0 | 0.008 | 0.798 | 0.231 | 0 | 0.200 |
| N51I+C59R | 0.977 | 0.944 | 0.733 | 0.750 | 0.179 | 0.836 | 0.490 | 0.789 | 0.400 | 1.000 | 0.370 | 0.918 | 0.905 | 0 | 0.606 | 0.933 | 0.952 | 0 | 0 |
| N51I+S108N | 0.991 | 0.944 | 0.867 | 0.982 | 0.154 | 0.909 | 0.525 | 0.779 | 0.371 | 1.000 | 0.389 | 0.916 | 0.905 | 0 | 0.606 | 0.933 | 0.947 | 0.214 | 0.714 |
| C59R+S108N | 0.986 | 1.000 | 0.867 | 0.768 | 0.205 | 0.836 | 0.713 | 0.853 | 0.371 | 1.000 | 0.944 | 0.996 | 1.000 | 0.909 | 0.971 | 1.000 | 0.995 | 0 | 0 |
| Triple Mutant* | 0.977 | 0.944 | 0.733 | 0.750 | 0.154 | 0.836 | 0.490 | 0.779 | 0.371 | 1.000 | 0.370 | 0.916 | 0.905 | 0 | 0.606 | 0.933 | 0.947 | 0 | 0 |
| Quadruple Mutant** | 0 | 0 | 0 | 0 | 0 | 0 | 0 | 0 | 0 | 0 | 0.167 | 0.422 | 0.811 | 0 | 0.010 | 0.752 | 0.203 | 0 | 0 |
| *crt* |  |  |  |  |  |  |  |  |  |  |  |  |  |  |  |  |  |  |  |
| K76T | 0 | 0.722 | 0.200 | 0.661 | 0.205 | 0.727 | 0.223 | 0.674 | 0.514 | 1.000 | 0.889 | 0.973 | 0.989 | 0.909 | 0.885 | 0.981 | 0.925 | 1.000 | 1.000 |
| Q271E | 0 | 0.722 | 0.200 | 0.643 | 0.205 | 0.727 | 0.233 | 0.663 | 0.657 | 1.000 | 0.907 | 0.941 | 0.979 | 0 | 0.885 | 0.986 | 0.914 | 0 | 0 |
| N326S | 0 | 0 | 0 | 0 | 0 | 0.018 | 0 | 0 | 0 | 0 | 0.241 | 0.647 | 0.989 | 0 | 0.115 | 0.952 | 0.358 | 0 | 0 |
| I356T | 0 | 0 | 0 | 0.196 | 0.026 | 0.636 | 0.015 | 0.126 | 0.229 | 0.250 | 0.833 | 0.672 | 0.989 | 0 | 0.115 | 0.990 | 0.380 | 0 | 0 |
| *kelch13* |  |  |  |  |  |  |  |  |  |  |  |  |  |  |  |  |  |  |  |
| K189T | 0.091 | 0.056 | 0.067 | 0.196 | 0.615 | 0.545 | 0.530 | 0.411 | 0.400 | 0.500 | 0.130 | 0 | 0.011 | 0 | 0 | 0 | 0 | 0.857 | 0.429 |
| K189N | 0.005 | 0 | 0 | 0 | 0.026 | 0.091 | 0.020 | 0 | 0.029 | 0 | 0 | 0 | 0 | 0 | 0 | 0 | 0 | 0 | 0 |
| Y493H | 0 | 0 | 0 | 0 | 0 | 0 | 0 | 0 | 0 | 0 | 0 | 0.087 | 0 | 0 | 0 | 0 | 0.021 | 0 | 0 |
| C580Y | 0 | 0 | 0 | 0 | 0 | 0 | 0 | 0 | 0 | 0 | 0 | 0.387 | 0.105 | 0 | 0 | 0.138 | 0.059 | 0 | 0 |
| *gch1* |  |  |  |  |  |  |  |  |  |  |  |  |  |  |  |  |  |  |  |
| No duplication | 0.032 | 1 | 1 | 0.534 | 1 | 0.909 | 0.658 | 0.547 | 0.971 | 1 | 0.852 | 0.954 | 0.926 | 1 | 0.990 | 0.852 | 0.893 | 1 | 1 |
| Promoter duplication | 0.968 | 0 | 0 | 0.446 | 0 | 0.091 | 0.292 | 0.453 | 0.011 | 0 | 0.037 | 0.002 | 0 | 0 | 0 | 0.010 | 0 | 0 | 0 |
| Whole gene duplication | 0 | 0 | 0 | 0.020 | 0 | 0 | 0.050 | 0 | 0 | 0 | 0.111 | 0.044 | 0.074 | 0 | 0.010 | 0.138 | 0.107 | 0 | 0 |

**dhfr* N51I, C59R & S108N haplotype. ** *dhfr* N51I, C59R, S108N & I164L haplotype; DRC Democratic Republic of Congo

**Additional Table 7:** Top hits for Malawi pairwise positive selection (XP-EHH) analysis.

| **Gene ID** | **Populations** | **XP-EHH**** | **Gene** |
| --- | --- | --- | --- |
| *PF3D7_0212500* | DRC | 6.511 | Conserved unknown |
| *PF3D7_0215300* | DRC, Ghana, Guinea | -6.087, -6.912, -6.872 | *acs8*; Acyl-CoA synthetase |
| *PF3D7_0307900* | DRC | -6.866 | Conserved unknown |
| *PF3D7_0321800* | Ghana | 6.802 | WD repeat-containing protein |
| *PF3D7_0416900* | Mali | -6.257 | Conserved unknown **(*near dhfr*)** |
| *PF3D7_0417400* | Colombia, Ghana | -6.184, -6.323 | Conserved unknown **(*near dhfr*)** |
| *PF3D7_0513200* | Laos | 6.037 | Conserved unknown |
| *PF3D7_0525100* | Ghana, Guinea | -6.337, -6.514 | *acs10*; Acyl-CoA synthetase |
| *PF3D7_0526600* | Laos, Mali | 7.018, -6.181 | Conserved unknown |
| *PF3D7_0529000* | Bangladesh, Laos, Myanmar | 7.162, 6.947, 6.245 | Conserved unknown |
| *PF3D7_0620400* | DRC, West Africa, Myanmar | 5.981, 11.079, 6.125 | *Msp10* |
| *PF3D7_0629700* | DRC | 7.241 | *Set1* |
| *PF3D7_0709100* | Bangladesh, DRC, Gambia, Southeast Asia | 7.529**,** 9.330, 7.759, 8.663 | *Cg1* protein **(near *crt*)** |
| *PF3D7_0709200* | Cambodia, Myanmar, Thailand | 6.327, 6.259, 6.095 | *GLP3* (Cg6 protein) **(near *crt*)** |
| *PF3D7_0709300* | Cambodia, DRC, Thailand, Vietnam | 6.759, 8.073, 7.229, 6.470 | *cg2* (near ***crt***) |
| *PF3D7_0709600* | DRC, Gambia | 8.498, 6.520 | *pop1* |
| *PF3D7_0710000* | Peru | 6.019 | Conserved unknown |
| *PF3D7_0810200* | Gambia | -6.132 | *ABCK1* |
| *PF3D7_0810600* | Guinea | -6.477 | ATP-dependent RNA helicase DBP1 |
| *PF3D7_0810800* | Colombia, Guinea, Laos, Vietnam | -6.257, -6.532, -6.257, -6.125 | ***dhps*** |
| *PF3D7_0810900* | Colombia | -6.257 | Conserved unknown (near ***dhps***) |
| *PF3D7_0926500* | Bangladesh | -6.266 | Conserved unknown |
| *PF3D7_1223400* | DRC, West Africa, Kenya | -10.114, -9.217,-7.852 | **near *gch1*** |
| *PF3D7_1223500* | DRC, Gambia, Ghana, Guinea, Kenya | -9.998, -6.640, -6.434, -7.130, -6.431 | **near *gch1*** |
| *PF3D7_1218300* | Ghana | 6.294 | *ap2mu* |
| *PF3D7_1227500* | DRC | -6.231 | *cyc2* |
| *PF3D7_1335800* | DRC | 6.679 | Conserved unknown |
| *PF3D7_1352900* | Cambodia, Colombia | 6.357, 6.498 | Exported unknown, fam-f protein |
| *PF3D7_1324300* | Gambia, Ghana, Guinea | 6.295, 6.722, 7.758 | Conserved unknown membrane |
| *PF3D7_1335900* | Colombia, DRC, Mali, Peru, Tanzania, Vietnam | 6.682, 6.178, -6.171, 6.662, 6.088, 8.256 | *trap* |
| *PF3D7_1421100* | West Africa | -9.650 | Conserved unknown |

Positive (negative) scores indicate relative selection in the non-Malawi (Malawi) population. Bold indicates genes with known associations with drug resistance. DRC Democratic Republic of Congo.
