# Supplementary material for: Characterizing the impact of sustained sulfadoxine/pyrimethamine use upon the Plasmodium falciparum population in Malawi
Source: Malar J. 2016 Nov 29;15:575. doi: 10.1186/s12936-016-1634-6 (PMC5129638; doi:10.1186/s12936-016-1634-6)

**Additional Figure 1: Clustering through Principal Components Analysis of the combined dataset.** Analysis is based on a pairwise Manhattan distance SNP matrix for all samples; **(A)** East Africa; (**B**) Africa; (**C,D**) Global. Colours indicate countries with point styles indicating continents. West Africa (n=430): Burkina Faso (BUF, 39), Gambia (GAM, 55), Ghana (GHA, 202), Guinea (GUI, 95), Mali (MAI, 35), Nigeria (NIG, 4); Central and East Africa (n=253): Dem. Rep. of Congo (DRC, 56), Kenya (KEN, 15), Malawi (MAW, 220), Tanzania (TAN, 18); South and South-East Asia (n=1187): Bangladesh (BAN, 54), Cambodia (CAM, 526), Laos (LAO, 104), Myanmar (MYA, 95), Papua New Guinea (PNG, 11), Thailand (THA, 210), Vietnam (VIE, 187); South America (n=21); Colombia (COL, 14), Peru (PER, 7).


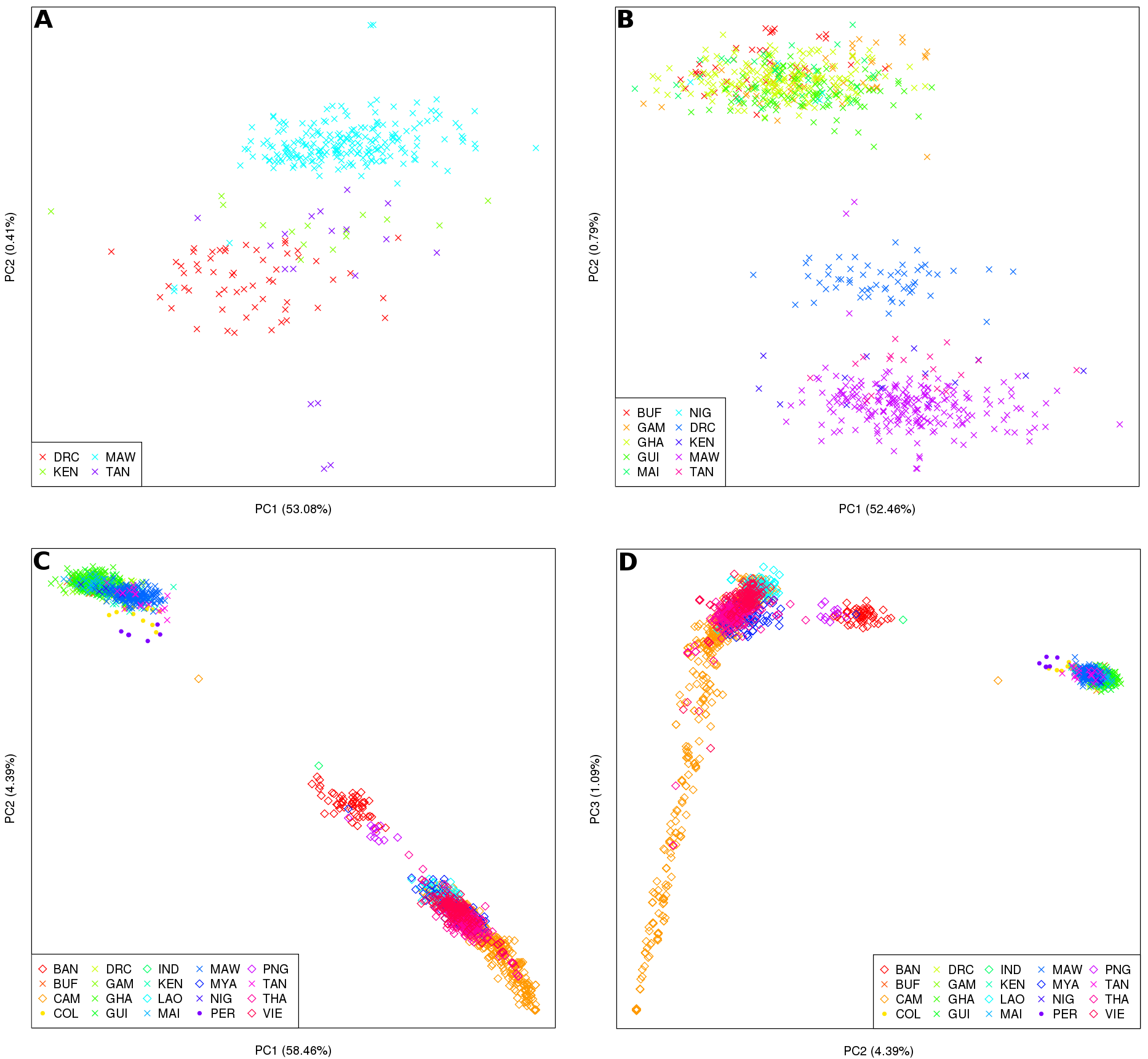


**Additional Figure 2: XP-EHH around *gch1* comparing the Malawi population with those from the DRC, Ghana and Guinea.** Negative values indicate relative fixation in Malawi, whilst positive values indicate relative fixation in the non-Malawian population. Green indicates *gch1* whilst the immediate grey bars indicate the same neighbouring genes as in Figure 2. In addition, the two upstream genes *Pf3D7_1223400* and *Pf3D7_1223500* with the most significant XP-EHH are highlighted in grey. Red lines indicate our significance threshold of absolute XP-EHH scores greater than 4.


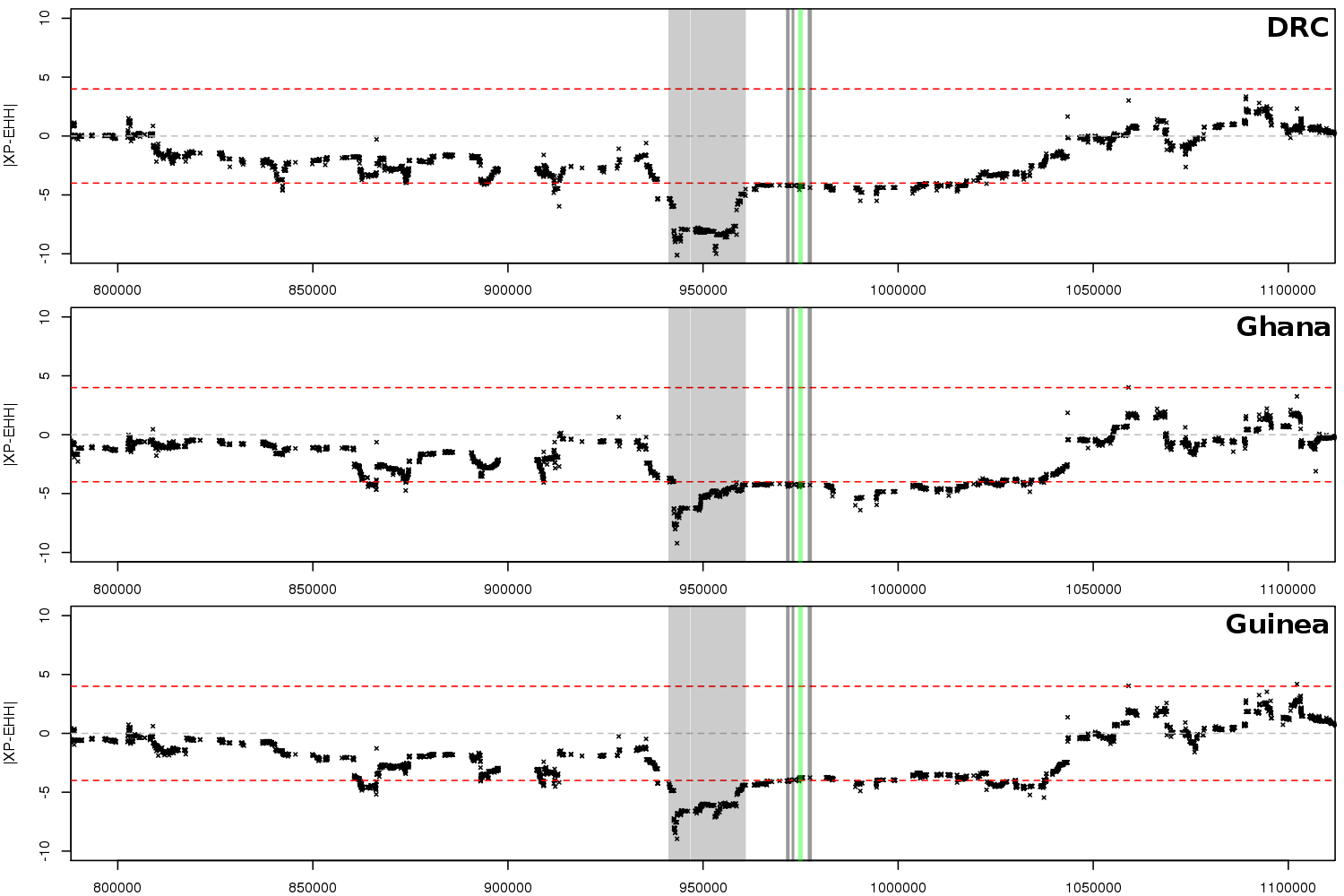


**Additional Figure 3: XP-EHH selection around *gch1* between duplication-positive and duplication-negative DRC, Ghana and Guinea populations.** Negative values indicate relative fixation in the duplication-positive population, whilst positive values indicate relative fixation in the duplication-negative population. Green indicates *gch1* whilst the immediate grey bars indicate the same neighbouring genes as in Figure 2. In addition, the two upstream genes *Pf3D7_1223400* and *Pf3D7_1223500* with the most significant XP-EHH are highlighted in grey. Red lines indicate our significance threshold of absolute XP-EHH scores greater than 4.


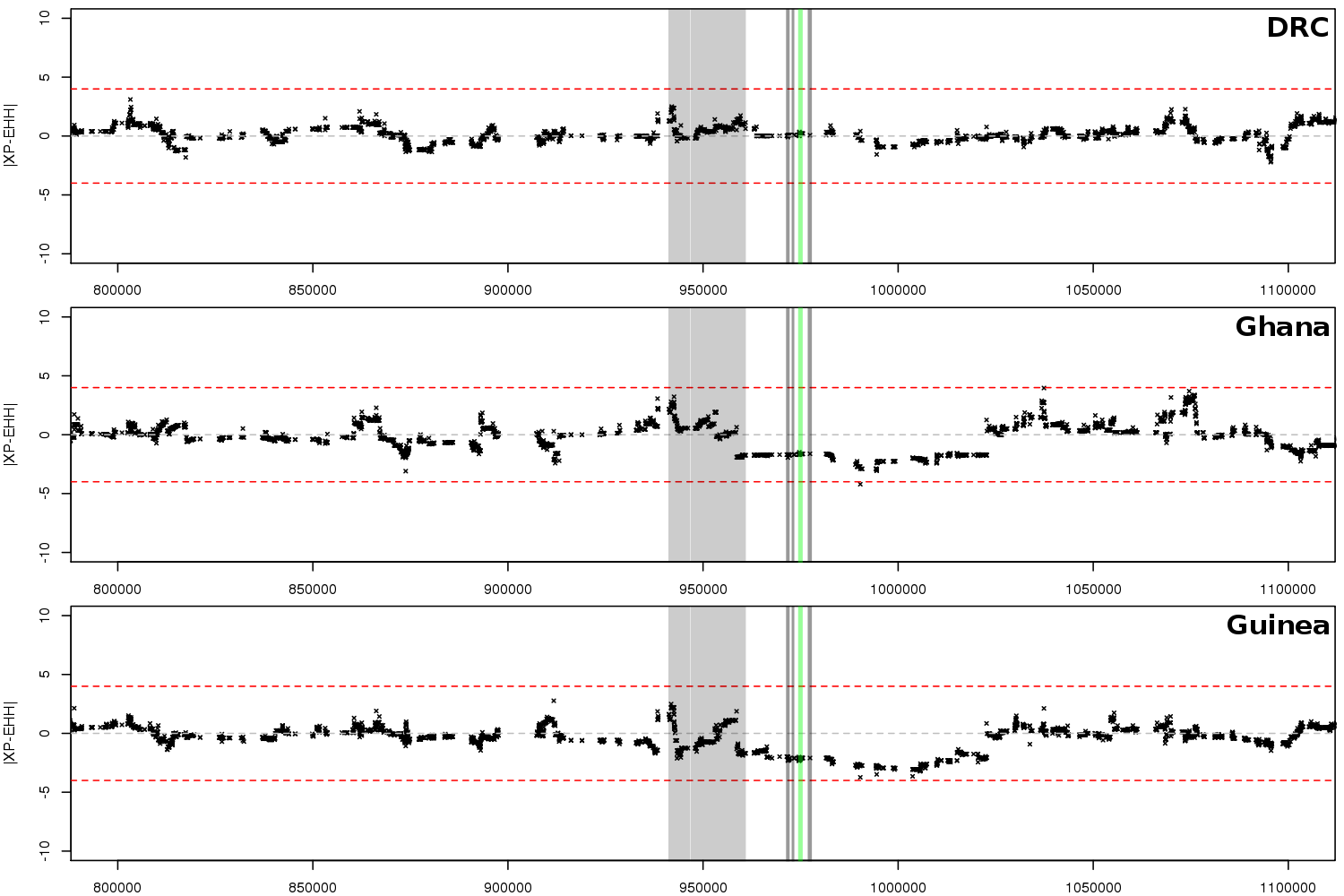


**Additional Figure 4: Coverage for the *gch1* region in five reference strains.** Whilst the previously identified *gch1* duplication is present within 3D7 (ERS009999), 7G8 (ERS016318), DD2 (ERS010540) and GB4 (ERS016319) and confirmed absent in HB3 (ERS010539), no reference sample was shown to contain the novel promoter duplication. Green indicates *gch1*, blue indicates the region for the most frequent promoter duplication, and grey indicates neighbouring genes. Coverage is scaled against the median coverage (indicated by the dashed red line) for chromosome 12 and calculated for 100bp windows, with an offset of 25bp.


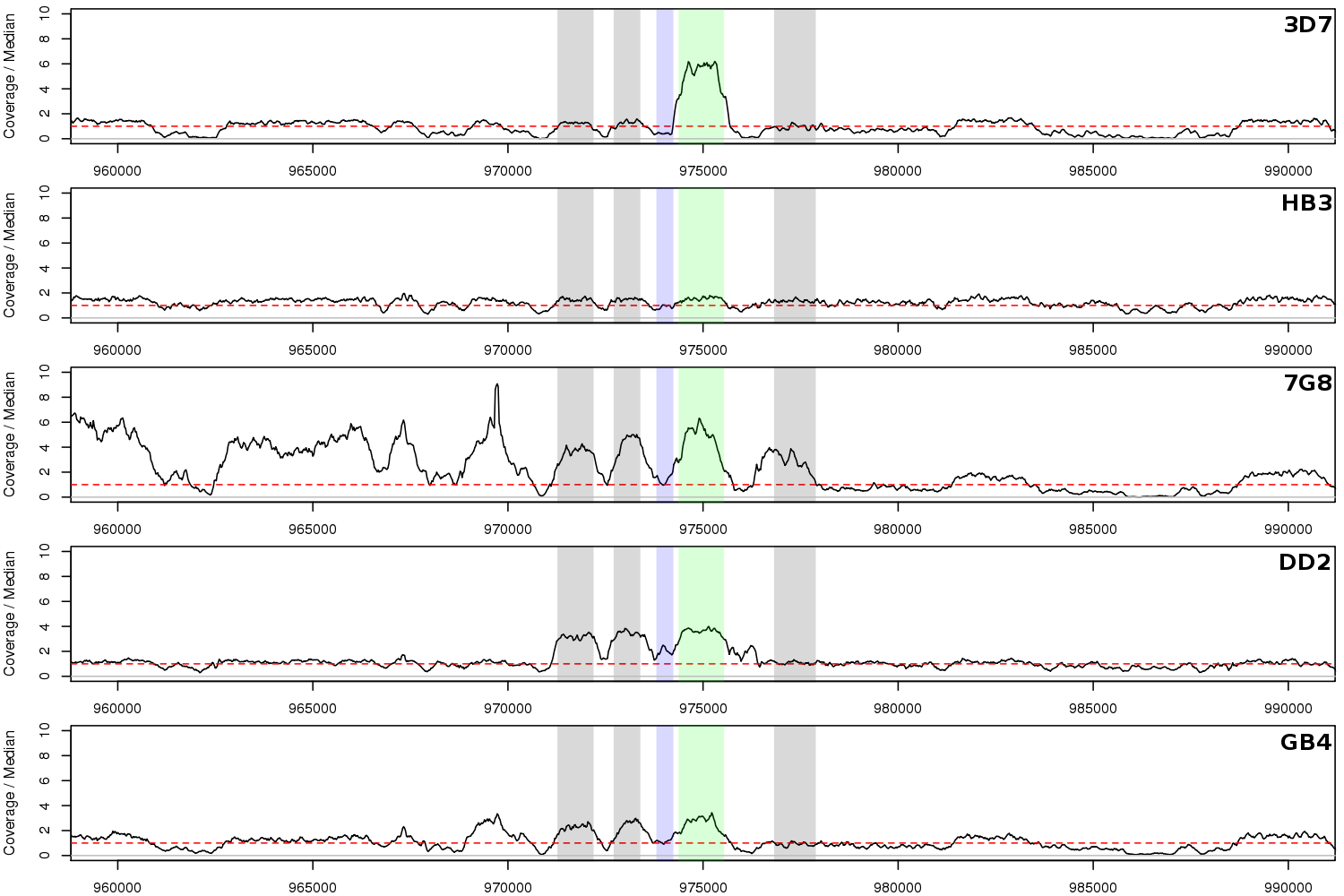

Supplement: Supplementary file 2 — Additional file 2. Additional figures. [file 12936_2016_1634_MOESM2_ESM.docx]
